# Sifting Through the Noise: Identifying Core Fundamentals of Healthy Eating Early in the Life Cycle

**DOI:** 10.1007/s13668-025-00675-8

**Published:** 2025-06-21

**Authors:** Diana Schnee, Christina DeTallo, Kadakkal Radhakrishnan, Senthilkumar Sankararaman

**Affiliations:** Department of Pediatric Gastroenterology, Hepatology, and Nutrition, Cleveland Clinic Children’s Hospital, Cleveland, OH 44195 USA

**Keywords:** Life cycle, Nutrition, Pregnancy, Neonate, Infancy, Malnutrition, Adolescent

## Abstract

**Purpose of Review:**

Optimal nutrition is important across all age groups. However, nutrition provision during early years of life is more crucial and any aberrations in this period could lead to long lasting consequences. The first 1000 days starting from conception to the second birthday is the period of maximum growth velocity and brain growth and suboptimal nutrition in this period should be avoided at all cost.

**Recent Findings:**

Feeding and nutritional optimization in the early stages of life provides a critical window of opportunity. Implementation of nutritional interventions can be challenging in this age group due to multiple factors such increased nutritional needs, environmental circumstances, and feeding difficulties.

**Summary:**

Interventions should focus on improving maternal/fetal, infant, child, and adolescent nutrition so that every child can reach her or his full potential of growth and development. Interventions should be simple, sustainable, socio-culturally acceptable and should begin in the fetal period.

## Introduction

Ensuring optimal feeding and provision of balanced nutrition during pregnancy, infancy, and young childhood is vital to establish successful growth and development of children to their maximum potential [1]. The first 1000 days starting from conception to the second birthday is the period of maximum growth and development and suboptimal nutrition in this critical period can lead to long-lasting impact [1]. In this review, we detail the fundamental concepts of healthy eating in early stages of life starting from maternal health through adolescence.

### Prenatal Period

Maternal nutritional status and maternal health has profound effects on fetal health. Even at conception, maternal nutrient deficiencies may have long-lasting consequences on the fetus. The increased demand for both macro-and micronutrients to generate life is taxing on the mother as the body will prioritize fetal demands. While the effects of prenatal malnutrition in the setting of famine showed increased risk for chronic disease in the offspring including development of obesity, cardiovascular disease, and type 2 diabetes later in life [2], the effects of poor nutrition despite food abundance in the resource-rich settings may be just as consequential.

Nutritional imbalances, including both inadequate or excessive maternal nutrient intake, can lead to abnormal gestational weight gain [3]. In turn, this can result in suboptimal fetal growth patterns potentially causing low birth weight, small-/large for gestation age, fetal growth restriction, or macrosomia [3]. These children may have increased risk for metabolism-related chronic diseases later in childhood and as adults [3]. On the other hand, a well-balanced maternal diet is associated with reduced risk for gestational diabetes, preterm birth, preeclampsia, and gestational hypertension. An optimal diet is defined as containing adequate quantities of lean protein, fruits, vegetables, whole grains, nuts, monounsaturated fats, legumes, and fiber [3]. Similarly, a diet containing saturated fat, simple sugars, red meat, and ultra-processed food is not recommended and these foods should be consumed in minimal quantities. Incorporation of low-mercury fish (as categorized by the United States Environmental Protection Agency) is also important for optimal neurocognitive development [4]. Most notably, a focus on the avoidance of refined carbohydrates and added sugars during pregnancy and postpartum (first 1000 days of life from conception) has recently shown to be associated with reduced risk for diabetes and hypertension in later life [5] Conditions such as maternal obesity, poorly controlled gestational diabetes, and poor quality of maternal diet may lead to fetal overnutrition. This can result in macrosomia or large for gestational age (LGA) as well as increased risk for overweight or obesity in the offspring [6].

Optimal intake of micronutrients is also vital for successful pregnancy outcomes and the well-established role of prenatal micronutrients are discussed here. Adequate consumption of folic acid in the periconception period is associated with reduction of neural tube defects [7]. Also, low maternal serum folate levels have been associated with poor fetal growth [7]. Pregnant women are at high risk of development of iron deficiency anemia due high fetal demand for iron often coupled with poor maternal iron intake. Maternal iron deficiency anemia has been associated with multiple adverse fetal outcomes including low birth weight (LBW) and prematurity [3, 7]. Choline and omega-3 fatty acid intake have been linked to improved fetal neurological development, and reduced risk of atopic conditions such as allergy, asthma, and eczema [3, 7]. Requirements for maternal iodine increase prenatally to support fetal thyroid function and cognitive fetal development [7]. For mothers who consume vegan/vegetarian diets or a diet which is not complete and balanced nutritionally, additional supplementation of B-complex vitamins and zinc may be warranted [8, 9]. Low maternal zinc status (less than 60 mcg/dL) during the third trimester is associated with low birth weight and fetal growth restriction [7]. Commercially available prenatal vitamins help to mitigate the adverse effects of these nutritional deficiencies and support nutritional intake both during gestation and in the postpartum period to support lactation and maternal health. Initiation of prenatal vitamins at least 2–3 months prior to conception and throughout lactation is recommended to help enhance maternal and fetal nutrition [3].

### Maternal Health

Pre-existing maternal health conditions including diabetes mellitus, hypertension and obesity can perpetuate adverse fetal growth. Maternal obesity can increase the risk for congenital abnormalities, spontaneous abortions, and intrauterine fetal death [3, 7]. Maternal fat mass and high serum triglyceride levels are primary drivers of fetal fat mass development and are associated with childhood obesity and development of inflammatory diseases such as metabolic dysfunction-associated fatty liver disease [3]. Pre-existing or gestational diabetes, particularly uncontrolled, can lead to accelerated fetal growth and increased fat deposition from increased fetal insulin. Further, increased fetal glucose levels with hyperinsulinemia results in LGA infants and predispose them to complications such as increased Caesarean section, traumatic delivery (including shoulder dystocia), and neonatal hypoglycemia [7]. The late complications of gestational diabetes include predisposition of offspring to development of obesity, asthma, and metabolic syndrome ([7]. Severe maternal diabetes with placental insufficiency can result in fetal growth restriction (FGR). FGR can have long term effects with increased potential for learning deficits in childhood and spastic cerebral palsy. Furthermore, FGR has been associated with increased frequency of chronic diseases in adulthood including such as systemic hypertension, ischemic heart disease, and type 2 diabetes. [7]. This association is referred to as the Barker’s (thrifty phenotype) hypothesis and believed to occur due to epigenetic modifications in the fetus in response to FGR [10, 11].

### Neonatal Period

Human milk is the preferred exclusive source of nutrition for normal infants, born at term, for at least the first six months of life. The American Academy of Pediatrics (AAP) breastfeeding guidelines in 2022 were updated to state that breast milk is recommended as a complimentary source of nutrition for toddlers two years old, and beyond as mutually desired. This was a change from the previous recommendations of providing breast milk for at least one year of age and now supports the World Health Organization (WHO) guidelines [12]. Breast milk contains immunoglobulins which is secreted in response to infants'saliva and act as a natural immune defense [13]. Breast milk is also antimicrobial and anti-inflammatory [14] Exclusive breastfeeding is associated with lower rates of respiratory tract infections, ear infections, severe diarrhea, and obesity [3, 14]. Breastfeeding is also associated with higher fruit and vegetable intake in offspring possibly due to the varied flavor profile of maternal milk [15]. The benefits of breastfeeding also extend to the mother. Breastfeeding longer than 12 months can reduce risk for type 2 diabetes, hypertension, breast cancer, and ovarian cancer in the mother [14].

Breast milk is relatively high in carbohydrates (50% of calories) and fat (40% of calories) and low in protein (10% of calories) [16]. Maternal diet should be enriched and balanced with all essential nutrients to prevent deficiencies in the offspring. The nutrient content of breast milk is a direct reflection of maternal dietary intake and nutrition status [3, 17]. Mother’s dietary intake of thiamin, riboflavin, vitamin B6, vitamin B12, and choline impacts the amount present in her breast milk and poor maternal status could result in poor infant stores [17]. Maternal supplementation of thiamin, riboflavin, vitamin B6, vitamin B12, choline, vitamin C, fat-soluble vitamins, iodine, and selenium also enhances their concentrations found in breast milk [17]. Especially for women who are following a vegan or vegetarian diet, it is important for adequate maternal status of vitamin B12 to prevent deficiency in the infant [18].

Latest studies recommend that maternal supplementation of vitamin K and vitamin D does (unless consumed at high doses) not impact nutritional stores in the infant and these nutrients should be individually supplemented at various points of infancy [19]. At birth, it is standard practice to provide one mg of vitamin K1 injection to prevent vitamin K deficient bleeding since infants are born with low levels of vitamin K [20]. Throughout infancy, vitamin D supplementation with 400 IU per day is recommended for any infants who are primarily breastfed or taking less than one liter of infant formula daily [21]. Alternatively, mothers can supplement with 6400 IU/day to supply breast milk with adequate vitamin D [19]. This is to prevent nutritional vitamin D deficiency and rickets and to support skeletal development [21].

While about 84% of mothers initiate breastfeeding, only about 25% are exclusively breastfeeding at 6 months [14]. Many women are faced with significant barriers to providing breast milk for their children including work demands, the physical demand of production, and in some cultures, a social stigma. For those unable to breastfeed, who opt not to, or whose maternal milk is not compatible with the infant, there are many commercially available dairy-based and soy-based infant formula options. Many products are designed and marketed to target common aliments of gastroesophageal reflux, fussiness, colicky pain, gassiness, and constipation with lack of stronger evidence. In cases of food protein intolerances, extensively hydrolyzed (or amino-acid based formulas for severe cases) may be warranted. As these products are fortified with iron, additional supplementation (discussed in the next section) may not be warranted. Additional vitamin D supplementation is recommended if the intake of formula is less that one liter daily.

### Infancy (Postnatal to 12 Months of Age)

Breast milk or formula is recommended in the early postnatal period and complimentary food introduction should occur no earlier than 4 months of age as it may be associated with increased weight gain and adiposity during infancy and later in life [22]. In infants who are exclusively breastfed, it is strongly recommended to wait until 6 months of age to start solids at which point nutrient demands start to exceed that provided by breast milk [18].In formula-fed infants, complimentary foods should not be introduced before 4 months of age and only when the infant is showing developmental readiness to accept complimentary foods [18]. Before initiation of solids, an infant should show have postural control and demonstrate interest in food. Methods of food introduction vary from texture advancement starting with purees, to initiation with soft table foods, a process known as baby-led weaning.

While baby-led weaning is not associated with an increased risk for choking [23], it is important for an infant to have substantial trunk control to prevent adverse events. Comparatively, traditional baby feeding methods have involved parent-guided feeding which may not support development of self-feeding skills as early as in baby-led weaning [24, 25]. Even families who are not eager to fully adopt baby-led weaning may still opt for a modified baby-led feeding [24]. This focuses on the infant initiating the act of eating by moving their mouth toward the feeding spoon or baby assisting parents with the spoon.

Infants who are breastfed should be offered iron-rich foods as first foods due to the low iron content of maternal milk and the expiration of fetal iron hepatic stores around 4–6 months of age [18, 26]. This could be from iron fortified infant cereals, meats, legumes, or beans and can be offered as purees or soft solids as in the case of baby led weaning. Comparatively, formula-feed infants do not have this high nutrient demand since formula is iron fortified (~ 12 mg/L) and can start with any food group. Additionally, iron supplementation at a dose of 1 mg/kg/day (dosing based on elemental iron) may be warranted to support stores in a breastfed, term infant, around 4 months of age, until iron-rich complimentary foods having equivalent iron content have been introduced [26]. Preterm infants who are breastfed should receive iron supplementation at dose of 2 mg/kg/day (dosing based on elemental iron) starting at one month of age and continued until iron-rich complimentary foods with equivalent iron content can be consumed [26]. Many families also opt to start with vegetables with the intention to limit an infant’s preference for sweetness and encourage vegetable preference in childhood [27]. Historically, it has been customary practice to wait three days between the successive introduction of new foods, however, there is no evidence support for this practice, and it can prolong the process of food and flavor exposure needed to support a well-balanced diet [28]. In theory, low allergenic foods could be introduced on consecutive days. Infants should be exposed to the nine common food allergens (cow milk, soy, egg, wheat, peanut, tree nuts, fish, crustacean shellfish, and sesame) to establish tolerance and prevent Ig-E mediated allergies. Specifically, recommendations support the introduction of peanut and egg around 6 months of age, but not prior to 4 months of age with the goal of repeated ingestion at least once per week [28, 29]. Maternal avoidance of allergens and/or use of hypoallergenic infant formula is not a recommended means of prevention of food allergies [28]. It is important for infants to be offered a wide variety of nutrient-dense foods, diverse flavors, and textures, especially during the first year of life [25]. This is an impressionable time, and limited exposure may be related to more selective eating later in life [30]. Parents should model balanced eating habits and incorporate children into familial mealtime practices.

Offering new foods repeatedly, even up to 15 times, may be required for an infant to accept a new food. Balanced nutrition early in life helps to set a proper foundation for growth and development [25]. Feeding can be challenging in infants and young children due to many factors such as behavioral feeding aversion, sensory processing issues, developmental delay, and medical disorders which can affect swallowing [31]. Studies have noted that nearly up to one-quarter of children may have feeding difficulties [32]. Early recognition of these difficulties and referral to multidisciplinary team (nutrition-focused pediatric physicians, registered dietitians, speech language pathologists, occupational therapists, behavioral psychologists, registered nurses, and social workers) [31].

At 1 year of age, children are screened for iron deficiency through hemoglobin analysis and for lead toxicity. Iron deficiency anemia, defined as a hemoglobin concentration less than 2 standard deviation (SD) below the mean for age and gender, can easily be treated through supplementation with and/or dietary changes and will help prevent long term effects including cognitive delays and poor growth [26]. Lead exposure with mildly elevation of blood lead level is treated through a diet high in iron, vitamin C, and calcium, along with further evaluation and elimination of the source of the lead exposure. High levels of lead may need chelation therapy.

### Childhood, Adolescence, and Beyond

Childhood continues to be a crucial time for optimal nutrition as it is the building block for growth and development. In acknowledgement of the importance of nutrition in the prenatal and early life period, the 2020–2025 USDA Dietary Guidelines were the first guidelines to include recommendations for prenatal nutrition and for infants and toddlers. These guidelines focus on balanced nutrition to prevent chronic disease in all stages of life while focusing on consumption of nutrient-dense foods to meet and not exceed energy demands. Nutrient dense foods include a wide variety of vegetables, whole fruit, whole grains, dairy or dairy alternatives, protein rich foods and healthy oils/fats. Additionally, guidelines support avoidance/limited intake of added sugar, saturated fat, sodium, and alcohol [18]. Intake of free sugar should be reduced with a desirable goal of < 5% of energy goal in children and adolescents between 2–18 years [33]. Sugar should be ideally consumed as part of main meal and in natural forms such as milk and other dairy products, and fresh fruits rather than fruit juices, sweetened sugar beverages, and smoothies [33]. Many parents elect to switch cow’s milk in children to other plant-based milk-substitutes such as almond milk, cashew milk, coconut milk, flax-seed milk, hemp milk, oat milk, pea milk, rice milk, and soy milk [34]. Many of these products have low quantity of proteins, fat and other nutrients and clinicians should educate the families regarding these facts [34].

**Fig. 1:**
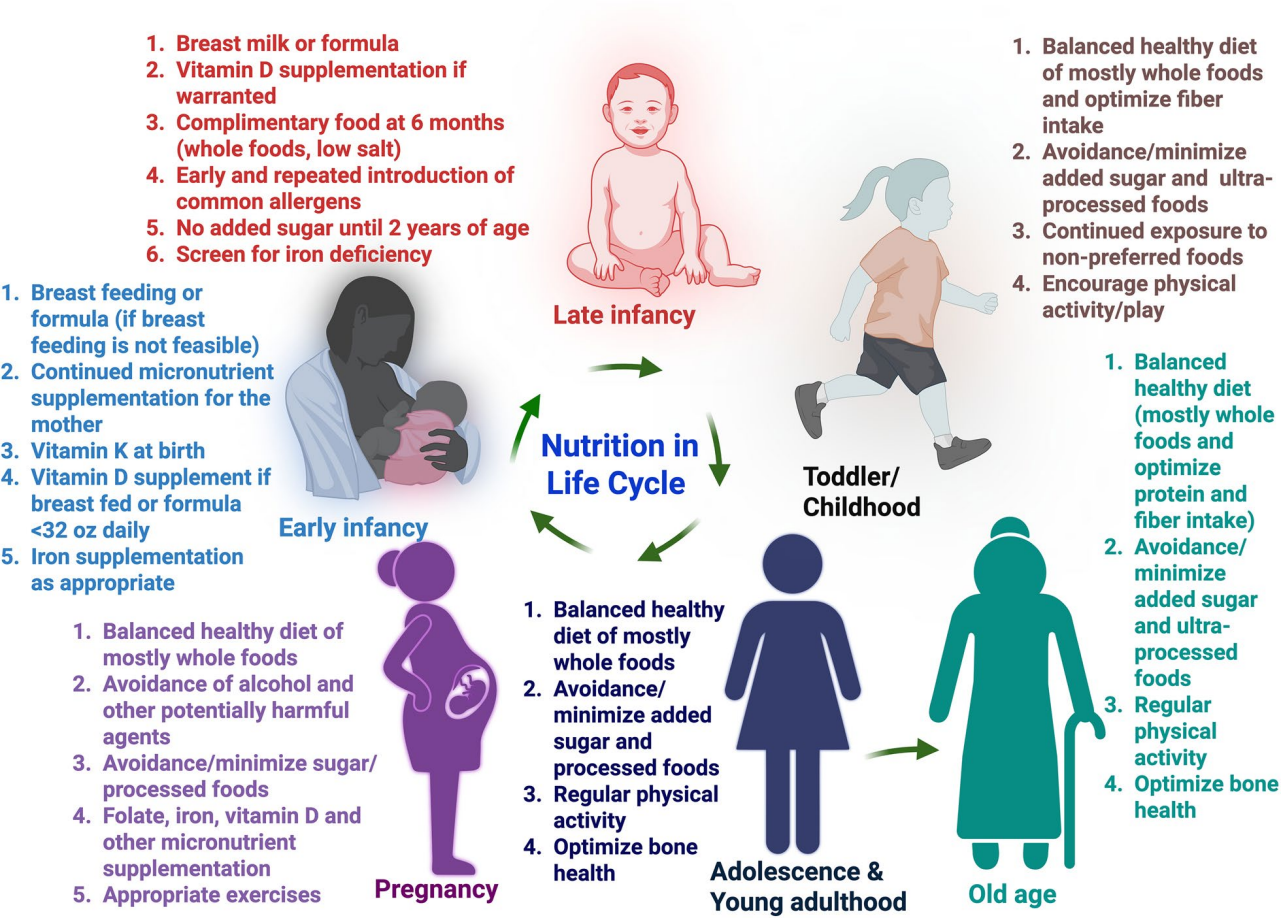
Basics of core nutrition principles and lifestyle recommendations during various stages of life cycle. Created with Biorender.com

Growth during adolescence is rapid and only second to that of infancy but often overlooked period of life [35]. This period has increased nutritional needs. A successful transition of adolescents to young adults and optimal nutritional intake during adolescence are closely interrelated [36]. In this age, eating pattern is highly influenced by peers, parental input, food factors (food availability, cost, quality, and preferences), body image, mass and social media, and individual and cultural beliefs [35, 36]. Poor nutrition during this period can predispose to both short- and long-term health effects such as poor bone health, risks of overweight/obesity, and increased predisposition to metabolic risk factors [35]. Along with optimal macro- and micronutrition, good daily routines such as regular physical activity, weight bearing exercises, adequate sleep, and encouragement towards a heath promoting behavior is recommended [35]. Routine calcium supplementation is not recommended for healthy children and teens, but calcium from dietary sources is encouraged to meet daily requirements [37]. The AAP recommends higher dietary allowance for vitamin D advised by the Institute of Medicine and reinforces evaluating vitamin D deficiency in pediatric population prone for increased bone fragility [37]. Routine testing for vitamin D deficiency in healthy children is not currently recommended due to lack of evidence to support this practice and also not cost effective [37]. The basics of core nutrition principles and lifestyle recommendations during various stages of life is depicted in Figure 1. Nutrition guidelines and recommendations from various national and international organizations and academic societies for general population, various age groups and diseases are listed in Table 1**.**

**Table 1:** List of websites detailing the nutrition recommendations encompassing for all age groups, and also focusing on certain disease states

Nutrition recommendations by various organizations	Website Uniform Resource Locator (URL)	References
The American College of Obstetrics and gynecology	https://www.acog.org/womens-health/faqs/nutrition-during-pregnancy	[38]
American Academy of Pediatrics	https://www.healthychildren.org https://www.aap.org/en/practice-management/bright-futures	[39] [40]
Academy of Nutrition and Dietetics	https://www.eatright.org	[41]
United States Department of Agriculture	https://www.usda.gov https://www.myplate.gov	[42] [43]
United States Department of Agriculture and Department of Health and Human Services Dietary Guidelines for Americans, 2020–2025	https://www.dietaryguidelines.gov/resources/2020-2025-dietary-guidelines-online-materials	[44]
Center for Disease Control and Prevention	https://www.cdc.gov/nutrition	[45]
World Health Organization	https://www.who.int/health-topics/nutrition	[12]
United Nations Children's Fund	https://data.unicef.org/topic/nutrition/child-nutrition	[46]
National Institute of Health	https://www.niddk.nih.gov/health-information/diet-nutrition	[47]
Harvard T.H.Chan School of Public Health	https://nutritionsource.hsph.harvard.edu	[48]
American Heart Association	https://www.heart.org/en/healthy-living/healthy-eating/eat-smart/nutrition-basics/aha-diet-and-lifestyle-recommendations	[49]
American Diabetic Association	https://diabetes.org/food-nutrition	[50]
U.S. Food and Drug Administration	https://www.fda.gov/food/nutrition-education-resources-materials/nutrition-facts-label	[51]

### Growth Faltering

Optimal growth is dependent on multiple things such as nutrition, genetic potential, hormones, and environmental factors. Accurate measurements of serial anthropometrics is essential in evaluating growth faltering. Weight-for-age, length/height-for-age, weight-for length (in children aged < 2 years), and body-mass-index (BMI, in children aged > 2 years) [52]. Growth faltering is more commonly seen in children less than two years of life. The other terms which are synonymously used for growth faltering include failure to thrive and growth failure [52–54].

Failure to thrive is often defined as decrease in 2 major centiles or growth below the fifth percentile [54]. Malnutrition (undernutrition) is referred to as an imbalance between nutrients needed by the body vs. intake which then results in cumulative deficits in macronutrients (energy or proteins) or micronutrients that may have negative outcomes in growth, development, and other outcomes [55]. Malnutrition is classified into mild, moderate, and severe based on single data points and also when multiple data points are available [54]. Malnourished children have adverse hospital outcomes such as increased mortality, higher length of stay, and increased hospital costs [56]. Also, pediatric malnutrition can lead to adverse outcomes such as loss of lean body mass, immune dysfunction and increased risk of infection, developmental delay, poor wound healing [55]. Many nutrition screening tools have been employed for hospitalized children and the common ones include the Screening Tool for Risk on Nutritional Status and Growth (STRONGkids), the Screening Tool for the Assessment of Malnutrition in Pediatric (STAMP), and the Pediatric Yorkhill Malnutrition Score (PYMS) [57]. Malnutrition can be classified as acute or chronic based on duration of less than or more than three months respectively. Once identified, malnutrition in pediatric age group can be best managed by interdisciplinary team consisting of physicians, dietitians, registered nurses social workers, feeding therapists, and behavioral psychologists. Table 2 details the etiology of mechanisms of nutrient imbalance resulting in malnutrition in pediatric patients.

**Table 2:** Details of duration, underlying conditions, and mechanisms of nutrient imbalance predisposing to pediatric malnutrition

Duration of Symptoms	Based on underlying illness	Mechanisms of nutrient imbalance resulting in malnutrition
Acute (< 3 months) Chronic (≥ 3 months)	No underlying illness Illness-related malnutrition - Infection - Inflammation - Trauma -Malignancy	**A. Reduced oral intake** 1. Famine and other calamities 2. Food insecurity 3. Psychosocial/neglect – poor maternal child bonding, maternal depression 4. Lactation disorders – reduced milk production and errors in latching technique 5. Inadvertent errors in mixing formula 6. Feeding disorders – oral aversion, texture issues, and forced feeding 7. Eating disorders – anorexia nervosa, ARFID 8. Secondary anorexia (related to illness such as inflammatory bowel disease, malignancy, HIV, tuberculosis, chronic pancreatitis, cardiac/hepatic/renal/pulmonary failure etc.) 9. Oropharyngeal dysphagia – cleft palate, conditions with hypotonia, aspiration of feeds, and severe neurological conditions 10. Esophagitis—gastroesophageal disease, and eosinophilic esophagitis 11. Gastritis – *Helicobacter pylori*
		B. **Increased nutrient loss** 1. Chronic vomiting (e.g. gastroesophageal disease, eosinophilic esophagitis, cyclic vomiting syndrome, pyloric stenosis, metabolic disorders, conditions causing increased intracranial tension) 2. Maldigestion – pancreatic disorders (cystic fibrosis), cholestatic diseases 3. Malabsorption – celiac disease, inflammatory bowel disease and other disorders of immune dysregulation, short bowel syndrome, congenial malabsorption disorders, SIBO, chronic graft vs host disease, giardiasis, and environmental enteropathy 4. Renal loss – diabetes mellitus, renal tubular acidosis, nephrotic syndrome 5. Skin loss – extensive dermatoses
		C. **Increased metabolic demands** 1. Prematurity and IUGR/SGA 2. Congenital heart disease, cardiac failure 3. Chronic pulmonary insufficiency, cystic fibrosis 4. Chronic renal disorders 5. Hyperthyroidism 6. Malignant disorders 7. Chronic infections (HIV, tuberculosis, recurrent UTI)
		D. **Impaired nutrient utilization** 1. Inborn errors of metabolism 2. Genetic/chromosomal conditions 3. IUGR/SGA

*ARFID* Avoidant restrictive food intake disorder, *HIV* Human immunodeficiency syndrome, *IUGR* Intrauterine growth restriction, *SGA* Small for gestational age, and *SIBO* Small intestinal bacterial overgrowth, *UTI* Urinary tract infections

Stunting is defined by WHO as height-for-age < −2 standard deviations (SD) from the median on the WHO growth chart [58]. Similarly, wasting is defined as weight-for-height < −2 SD and underweight is defined as weight-for-age < −2 SD from the median values on the WHO growth chart [58]. in most instances, stunting results from chronic or recurrent undernutrition either in-utero and/or in early childhood [46, 58]. Children with stunting may not reach their full potential height and also their full cognitive potential [46]. Evidences support that children with stunting will have less school years, learning challenges and earn less as adults [46]. They also at risk of development of overweight and obesity later in life. Wasting is characterized by profound decline in nutritional status over a short period of time. Children with wasting have reduced immunity, increased susceptibility to infections and if not promptly managed will have increased mortality [46].

### Overweight/Obesity

Overconsumption of ultra-processed foods can easily disrupt optimal nutritional balance even in those who are active and may be contributing to the rising prevalence of obesity [59, 60]. Ultra-processed foods are defined as often chemically modified foods with added flavors, colors, and emulsifiers that are made into ready-to-eat, hyper-palatable food and drink products that are profoundly appealing and inherently unhealthy [61]. Ultra-processed foods make up more than half of the energy consumed in the United States and the United Kingdom [62]. Those foods make it simple to quickly consume a significant amount of added fat, salt, and sugar which is readily stored by the body in cases of an energy surplus. In a cohort study involving approximately 30,000 adults, ingestion of processed meat, unprocessed red meat, or poultry was significantly associated with cardiovascular disease but fish consumption was not [63]. Further, experts demonstrated that ingestion of processed meat or unprocessed red meat was associated with all-cause mortality but ingestion of poultry or fish did not have similar association [60].

Most recent data suggests that the prevalence of obesity is on the rise with 19.7% of children and 41.7% of adults being classified as obese between 2017 – March 2020 [64]. Additionally, there has been support for worsening obesity rates following the SARS-COVID-19 pandemic, as individuals may have experienced decreased physical activity, changes in meal patterning/increased snacking and possible increased consumption of ultra-processed foods. These behaviors may have contributed to energy imbalance and excessive weight gain [65, 66]. With children of overweight or obese parents being 1.97 times more likely to be overweight or obese than children with healthy-weight parents [67], the multigenerational effects of excessive nutrition are multiplying. Along with diet, changes in life style targeting increased physical activity, adequate sleep, and minimizing screen time are important interventions that need to implemented for a successful weight reduction strategy.

Since obesity is associated with chronic disease throughout the lifespan, this is an area of public health and nutrition that requires ongoing attention. To combat rising rates of obesity, there is a call to return to the roots of eating real food, whole foods, and minimally processed foods in all ages and stages of life. Meals must be home-cooked as much as possible and consumed as part of a community or with entire family. Instead of fast food, consumption of slow food, food that takes time to make and takes time to eat, must be encouraged. While notably a challenge in today’s face-paced, on-demand culture, this is crucial to break the cycle of obesity.

### Child Athletes

Athletics, exercise, and play are all required for optimal health at various levels throughout the age-span. Particularly, a focus on well-balanced and adequate nutrition is essential for child and adolescent athletes to support athletic performance and growth simultaneously. Protein helps with muscle repair and allows adaptation to exercise-induced stress, thereby allowing muscles to grow stronger. While there are no specific protein recommendations for child athletes, it has been noted that needs are likely higher than non-active peers [68]. This is a result of higher activity level leading to an increase in breakdown of muscle tissues and thereby a higher demand for dietary protein to build and repair the muscle [68]. Protein intake should primarily come from complete sources such as meat, fish, poultry, and eggs. However, in the case of a vegan/vegetarian diet, complete proteins (containing all 9 amino acids) can be made from a combination of whole grains plus legumes or beans. Protein needs increase by about 15–20% in vegan diets, regardless of activity level, due to low bioavailability of the protein sources [69]. Adequate intake of carbohydrates both pre- and post-athletics helps ensure optimal glycogen storage in muscles which are the first line of fuel during exercise [70]. Intake of healthy fats such as fats from nuts, avocado, and fish supports hormone function and aid in recovery [71]. Additionally, it is important that iron levels are adequate, especially in female athletes, as iron plays a key role in transporting oxygen throughout the body and metabolizing energy [71]. Child athletes should be encouraged to focus on high dietary quality, consumption of whole foods, and avoiding added sugars similar to their less-active peers.

## Conclusions

Nutrition plays a crucial role in the growth and development of children and suboptimal nutrient provision can have long lasting consequences. The first 1000 days starting from conception to the second birthday is the most crucial period for maximum growth velocity and brain growth. Strategies such as early recognition of dietary imbalance during pregnancy, infancy, childhood, and adolescence and successful nutritional optimization during these critical periods can prevent long-term adverse outcomes in growth and neurological outcome.

## Key References

- Gracner T, Boone C, Gertler PJ. Exposure to sugar rationing in the first 1000 days of life protected against chronic disease. Science. 2024;386(6725):1043–8.
  - In this research article, the association between sugar exposure in early life and chronic diseases later in life were explored in detail. It details how the limitation of exposure to sugar in the first 1000 days of life protects against chronic metabolic disease in adulthood.
- Wang Y, Wang K, Du M, Khandpur N, Rossato SL, Lo C-H, et al. Maternal consumption of ultra-processed foods and subsequent risk of offspring overweight or obesity: results from three prospective cohort studies. Bmj. 2022;379.
  - This article is a population-based prospective cohort study exploring the impact of consumption of ultra-processed foods during pregnancy on the offspring.
- Marshall NE, Abrams B, Barbour LA, Catalano P, Christian P, Friedman JE, et al. The importance of nutrition in pregnancy and lactation: lifelong consequences. American journal of obstetrics and gynecology. 2022;226(5):607–32.
  - In this article, the importance of improving women’s nutrition before, during, and after pregnancy to improve the health of woman and her offspring is emphasized.

## Data Availability

No datasets were generated or analysed during the current study.

## References

[1] WHO-Publication. 2009 [cited 2025 March 30]. Available from: https://www.who.int/publications/i/item/9789241597494.

[2] Lumey LH, Stein AD, Susser E. Prenatal famine and adult health. Annu Rev Public Health. 2011;32(1):237–62. 21219171 10.1146/annurev-publhealth-031210-101230 PMC3857581

[3] Marshall NE, Abrams B, Barbour LA, Catalano P, Christian P, Friedman JE, et al. The importance of nutrition in pregnancy and lactation: lifelong consequences. Am J Obstet Gynecol. 2022;226(5):607–32. 34968458 10.1016/j.ajog.2021.12.035 PMC9182711

[4] Bramante CT, Spiller P, Landa M. Fish consumption during pregnancy: an opportunity, not a risk. JAMA Pediatr. 2018;172(9):801–2. 30039174 10.1001/jamapediatrics.2018.1619 PMC7346675

[5] Gracner T, Boone C, Gertler PJ. Exposure to sugar rationing in the first 1000 days of life protected against chronic disease. Science. 2024;386(6725):1043–8. 39480913 10.1126/science.adn5421 PMC12238948

[6] Wang Y, Wang K, Du M, Khandpur N, Rossato SL, Lo C-H, et al. Maternal consumption of ultra-processed foods and subsequent risk of offspring overweight or obesity: results from three prospective cohort studies. Bmj. 2022;379 10.1136/bmj-2022-071767 PMC9533299 36198411

[7] Jain S, Maheshwari A, Jain SK. Maternal nutrition and fetal/infant development. Clin Perinatol. 2022;49(2):313–30. 35659089 10.1016/j.clp.2022.02.005

[8] Behere RV, Deshmukh AS, Otiv S, Gupte MD, Yajnik CS. Maternal vitamin B12 status during pregnancy and its association with outcomes of pregnancy and health of the offspring: a systematic review and implications for policy in India. Front Endocrinol. 2021;12:619176. 10.3389/fendo.2021.619176 PMC8074968 33912132

[9] Hovdenak N, Haram K. Influence of mineral and vitamin supplements on pregnancy outcome. Eur J Obstetrics Gynecol Reprod Biol. 2012;164(2):127–32. 10.1016/j.ejogrb.2012.06.020 22771225

[10] De Boo HA, Harding JE. The developmental origins of adult disease (Barker) hypothesis. Aust N Z J Obstet Gynaecol. 2006;46(1):4–14. 16441686 10.1111/j.1479-828X.2006.00506.x

[11] Jebasingh F, Thomas N. Barker hypothesis and metabolic syndrome. Metabolic Syndrome: Elsevier; 2024. p. 85–91.

[12] WHO. World Health Organization 2025 [Available from: https://www.who.int/health-topics/nutrition.

[13] Al-Shehri SS, Knox CL, Liley HG, Cowley DM, Wright JR, Henman MG, et al. Breastmilk-saliva interactions boost innate immunity by regulating the oral microbiome in early infancy. PLoS ONE. 2015;10(9):e0135047. 26325665 10.1371/journal.pone.0135047 PMC4556682

[14] Meek JY, Noble L, Breastfeeding So. Policy statement: breastfeeding and the use of human milk. Pediatrics. 2022;150(1):e2022057988. 10.1542/peds.2022-057988 35921640

[15] Beckerman JP, Slade E, Ventura AK. Maternal diet during lactation and breast-feeding practices have synergistic association with child diet at 6 years. Public Health Nutr. 2020;23(2):286–94. 31290381 10.1017/S1368980019001782 PMC6952591

[16] Kim SY, Yi DY. Components of human breast milk: from macronutrient to microbiome and microRNA. Clin Experimental Pediatrics. 2020;63(8):301. 10.3345/cep.2020.00059 PMC7402982 32252145

[17] Dror DK, Allen LH. Overview of nutrients in human milk. Adv Nutr. 2018;9:278S-S294. 29846526 10.1093/advances/nmy022 PMC6008960

[18] USDA. Scientific Report of the 2025 Dietary Guidelines Advisory Committee [Available from: https://www.dietaryguidelines.gov/2025-advisory-committee-report.

[19] Hollis BW, Wagner CL, Howard CR, Ebeling M, Shary JR, Smith PG, et al. Maternal versus infant vitamin D supplementation during lactation: a randomized controlled trial. Pediatrics. 2015;136(4):625–34. 26416936 10.1542/peds.2015-1669 PMC4586731

[20] CDC. https://www.cdc.gov/vitamin-k-deficiency/fact-sheet/index.html#:~:text=Vitamin%20K%20is%20needed%20for,brain%20damage%20and%20even%20death

[21] Wagner CL, Greer FR, Breastfeeding So, Nutrition Co. Prevention of rickets and vitamin D deficiency in infants, children, and adolescents. Pediatrics. 2008;122(5):1142–52. 10.1542/peds.2008-1862 18977996

[22] English LK, Obbagy JE, Wong YP, Butte NF, Dewey KG, Fox MK, et al. Timing of introduction of complementary foods and beverages and growth, size, and body composition: a systematic review. Am J Clin Nutr. 2019;109:935S-S955. 30982863 10.1093/ajcn/nqy267

[23] Fangupo LJ, Heath A-LM, Williams SM, Erickson Williams LW, Morison BJ, Fleming EA, et al. A baby-led approach to eating solids and risk of choking. Pediatrics. 2016;138(4). 10.1542/peds.2016-0772 27647715

[24] Arslan N, Kurtuncu M, Turhan PM. The effect of baby-led weaning and traditional complementary feeding trainings on baby development. J Pediatr Nurs. 2023;73:196–203. 37714048 10.1016/j.pedn.2023.09.006

[25] Lutter CK, Grummer-Strawn L, Rogers L. Complementary feeding of infants and young children 6 to 23 months of age. Nutr Rev. 2021;79(8):825–46. 33684940 10.1093/nutrit/nuaa143

[26] Baker RD, Greer FR, Nutrition Co. Diagnosis and prevention of iron deficiency and iron-deficiency anemia in infants and young children (0–3 years of age). Pediatrics. 2010;126(5):1040–50. 10.1542/peds.2010-2576 20923825

[27] Rapson JP, von Hurst PR, Hetherington MM, Mazahery H, Conlon CA. Starting complementary feeding with vegetables only increases vegetable acceptance at 9 months: a randomized controlled trial. Am J Clin Nutr. 2022;116(1):111–21. 35679432 10.1093/ajcn/nqac080 PMC9257464

[28] Fleischer DM, Chan ES, Venter C, Spergel JM, Abrams EM, Stukus D, et al. A consensus approach to the primary prevention of food allergy through nutrition: guidance from the American Academy of Allergy, Asthma, and Immunology; American College of Allergy, Asthma, and Immunology; and the Canadian Society for Allergy and Clinical Immunology. J Allergy Clin Immunol Pract. 2021;9(1):22–43. e4. 10.1016/j.jaip.2020.11.002 33250376

[29] Du Toit G, Roberts G, Sayre PH, Plaut M, Bahnson HT, Mitchell H, et al. Identifying infants at high risk of peanut allergy: the Learning Early About Peanut Allergy (LEAP) screening study. Journal of Allergy and Clinical Immunology. 2013;131(1):135–43. e12. 10.1016/j.jaci.2012.09.015 23174658

[30] De Cosmi V, Scaglioni S, Agostoni C. Early taste experiences and later food choices. Nutrients. 2017;9(2):107. 28165384 10.3390/nu9020107 PMC5331538

[31] Bruns DA, Thompson SD. Feeding challenges in young children: Toward a best practices model. Infants Young Child. 2010;23(2):93–102.

[32] Benjasuwantep B, Chaithirayanon S, Eiamudomkan M. Feeding problems in healthy young children: prevalence, related factors and feeding practices. Pediatric reports. 2013;5(2):38. 23904965 10.4081/pr.2013.e10 PMC3718228

[33] Mis NF, Braegger C, Bronsky J, Campoy C, Domellöf M, Embleton ND, et al. Sugar in infants, children and adolescents: a position paper of the European society for paediatric gastroenterology, hepatology and nutrition committee on nutrition. J Pediatr Gastroenterol Nutr. 2017;65(6):681–96. 28922262 10.1097/MPG.0000000000001733

[34] Merritt RJ, Fleet SE, Fifi A, Jump C, Schwartz S, Sentongo T, et al. North American Society for Pediatric Gastroenterology, Hepatology, and Nutrition position paper: plant-based milks. J Pediatr Gastroenterol Nutr. 2020;71(2):276–81. 32732790 10.1097/MPG.0000000000002799

[35] Heslin AM, McNulty B. Adolescent nutrition and health: characteristics, risk factors and opportunities of an overlooked life stage. Proceedings of the Nutrition Society. 2023;82(2):142–56. 36924388 10.1017/S0029665123002689

[36] Das JK, Salam RA, Thornburg KL, Prentice AM, Campisi S, Lassi ZS, et al. Nutrition in adolescents: physiology, metabolism, and nutritional needs. Ann N Y Acad Sci. 2017;1393(1):21–33. 28436102 10.1111/nyas.13330

[37] Golden NH, Abrams SA, Nutrition Co, Daniels SR, Abrams SA, Corkins MR, et al. Optimizing bone health in children and adolescents. Pediatrics. 2014;134(4):e1229-e43. 10.1542/peds.2014-2173 25266429

[38] ACOG. ACOG guidelines 2025 [Available from: https://www.acog.org/womens-health/faqs/nutrition-during-pregnancy.

[39] AAP. Healthy children 2025 [Available from: https://www.healthychildren.org.

[40] AAP. AAP Bright Futures 2025 [Available from: https://www.aap.org/en/practice-management/bright-futures.

[41] AND. Academy of Nutrition and Dietitics 2025 [Available from: https://www.eatright.org.

[42] USDA. US Deparmeent of Agriculture 2025 [Available from: https://www.usda.gov.

[43] USDA. MyPlate 2025 [Available from: https://www.myplate.gov.

[44] USDA.. United States Department of Agriculture and Department of Health and Human Services Dietary Guidelines for Americans, 2020–2025 2025 [Available from: https://www.dietaryguidelines.gov/resources/2020-2025-dietary-guidelines-online-materials.

[45] CDC. Center for Disease Control and Prevention 2025 [Available from: https://www.cdc.gov/nutrition.

[46] UNICEF. United Nations Children’s Fund 2025 [Available from: https://data.unicef.org/topic/nutrition/child-nutrition.

[47] NIH. Naational Institute of Health 2025 [Available from: https://www.niddk.nih.gov/health-information/diet-nutrition.

[48] Harvard. Harvard T.H.Chan School of Public Health 2025 [Available from: https://nutritionsource.hsph.harvard.edu.

[49] AHA. American Heart Association 2025 [Available from: https://www.heart.org/en/healthy-living/healthy-eating/eat-smart/nutrition-basics/aha-diet-and-lifestyle-recommendations.

[50] ADA. Ameerican Diabetes Association 2025 [Available from: https://diabetes.org/food-nutrition.

[51] FDA US. U.S. Food and Drug Administration 2025 [cited 2025 March 17]. Available from: https://www.fda.gov/food/nutrition-education-resources-materials/nutrition-facts-label.

[52] Tang MN, Adolphe S, Rogers SR, Frank DA. Failure to thrive or growth faltering: Medical, developmental/behavioral, nutritional, and social dimensions. Pediatr Rev. 2021;42(11):590–603. 34725219 10.1542/pir.2020-001883

[53] Cooke R, Goulet O, Huysentruyt K, Joosten K, Khadilkar AV, Mao M, et al. Catch-up growth in infants and young children with faltering growth: expert opinion to guide general clinicians. J Pediatr Gastroenterol Nutr. 2023;77(1):7–15. 36976274 10.1097/MPG.0000000000003784 PMC10259217

[54] Becker PJ, Carney LN, Corkins MR, Monczka J, Smith E, Smith SE, et al. Consensus statement of the Academy of Nutrition and Dietetics/American Society for Parenteral and Enteral Nutrition: indicators recommended for the identification and documentation of pediatric malnutrition (undernutrition). J Acad Nutr Diet. 2014;114(12):1988–2000. 25458748 10.1016/j.jand.2014.08.026

[55] Mehta NM, Corkins MR, Lyman B, Malone A, Goday PS, Carney L, et al. Defining pediatric malnutrition: a paradigm shift toward etiology-related definitions. J Parenter Enter Nutr. 2013;37(4):460–81. 10.1177/0148607113479972 23528324

[56] de Souza MF, Leite HP, Nogueira PCK. Malnutrition as an independent predictor of clinical outcome in critically ill children. Nutrition. 2012;28(3):267–70. 21872433 10.1016/j.nut.2011.05.015

[57] Fachal CV, Fernández-González SM, Moreno-Álvarez A, Solar-Boga A. Nutritional Screening Tools in the Pediatric Population: A Systematic Review. Nutrients. 2025;17(3):433. 39940291 10.3390/nu17030433 PMC11820693

[58] WHO-Definition. Definition for wasting, stunting and underweight 2025 [Available from: https://www.who.int/data/nutrition/nlis/info/malnutrition-in-children.

[59] Rauber F, Chang K, Vamos EP, da Costa Louzada ML, Monteiro CA, Millett C, et al. Ultra-processed food consumption and risk of obesity: a prospective cohort study of UK Biobank. Eur J Nutr. 2021;60:2169–80. 33070213 10.1007/s00394-020-02367-1 PMC8137628

[60] Poti JM, Braga B, Qin B. Ultra-processed food intake and obesity: what really matters for health—processing or nutrient content? Curr Obes Rep. 2017;6:420–31. 29071481 10.1007/s13679-017-0285-4 PMC5787353

[61] Monteiro CA, Cannon G, Levy RB, Moubarac J-C, Louzada ML, Rauber F, et al. Ultra-processed foods: what they are and how to identify them. Public Health Nutr. 2019;22(5):936–41. 30744710 10.1017/S1368980018003762 PMC10260459

[62] Marino M, Puppo F, Del Bo’ C, Vinelli V, Riso P, Porrini M, et al. A systematic review of worldwide consumption of ultra-processed foods: findings and criticisms. Nutrients. 2021;13(8):2778. 34444936 10.3390/nu13082778 PMC8398521

[63] Zhong VW, Van Horn L, Greenland P, Carnethon MR, Ning H, Wilkins JT, et al. Associations of processed meat, unprocessed red meat, poultry, or fish intake with incident cardiovascular disease and all-cause mortality. JAMA Intern Med. 2020;180(4):503–12. 32011623 10.1001/jamainternmed.2019.6969 PMC7042891

[64] Stierman B, Afful J, Carroll MD, Chen T-C, Davy O, Fink S, et al. National Health and Nutrition Examination Survey 2017-March 2020 prepandemic data files-development of files and prevalence estimates for selected health outcomes. National health statistics reports. 2021(158). 10.15620/cdc:106273. 10.15620/cdc:106273 PMC11513744 39380201

[65] Nour TY, Altintaş KH. Effect of the COVID-19 pandemic on obesity and its risk factors: A systematic review. BMC Public Health. 2023;23(1):1018. 37254139 10.1186/s12889-023-15833-2 PMC10227822

[66] Ferentinou E, Koutelekos I, Pappa D, Manthou P, Dafogianni C, PAPPA D, et al. The Impact of the COVID-19 Pandemic on Childhood Obesity: A Review. Cureus. 2023;15(9). 10.7759/cureus.45470 PMC10583857 37859918

[67] Lee JS, Jin MH, Lee HJ. Global relationship between parent and child obesity: a systematic review and meta-analysis. Clin Exp Pediatrics. 2021;65(1):35. 10.3345/cep.2020.01620 PMC8743427 33781054

[68] Hudson JL, Baum JI, Diaz EC, Børsheim E. Dietary protein requirements in children: methods for consideration. Nutrients. 2021;13(5):1554. 34063030 10.3390/nu13051554 PMC8147948

[69] Melina V, Craig W, Levin S. Position of the academy of nutrition and dietetics: vegetarian diets. J Acad Nutr Diet. 2016;116(12):1970–80. 27886704 10.1016/j.jand.2016.09.025

[70] Purcell LK, Society CP, Sports P, Section EM. Sport nutrition for young athletes.Paediatrics & child health. 2013;18(4):200-2. 10.1093/pch/18.4.200 PMC3805623 24421690

[71] Berg EK. Performance nutrition for the adolescent athlete: a realistic approach. LWW; 2019. p. 345–52. 10.1097/JSM.0000000000000744 31460947

